# Evolution of Nutritional Status in Patients Undergoing Autologous and Allogeneic Hematopoietic Cell Transplantation or CAR-T Therapy: A Retrospective Observational Study

**DOI:** 10.3390/cancers17010079

**Published:** 2024-12-30

**Authors:** Roberto Regazzoni, Sergio Ferrante, Emanuela Morenghi, Diego Lopane, Manuela Pastore, Daniela Cattani, Simone Cosmai, Francesco Colotta, Elena Azzolini, Marco Sguanci, Giovanni Cangelosi, Luca Cozzaglio, Beatrice Mazzoleni, Stefano Mancin

**Affiliations:** 1 Department of Biomedical Sciences, Humanitas University, Via Rita Levi Montalcini 4, Pieve Emanuele, 20090 Milan, Italy; roberto.regazzoni@st.hunimed.eu (R.R.); emanuela.morenghi@humanitas.it (E.M.); diego.lopane@hunimed.eu (D.L.); daniela.cattani@humanitas.it (D.C.); simone.cosmai@hunimed.eu (S.C.); francesco.colotta@humanitasresearch.it (F.C.); luca.cozzaglio@humanitas.it (L.C.); beatrice.mazzoleni@hunimed.eu (B.M.); 2 IRCCS Humanitas Research Hospital, Via Manzoni 56, Rozzano, 20089 Milan, Italymanuela.pastore@humanitas.it (M.P.); 3 A.O. Polyclinic San Martino Hospital, Largo R. Benzi 10, 16132 Genova, Italy; marco.sguanci@unicampus.it; 4 Unit of Diabetology, Asur Marche—Area Vasta 4 Fermo, 63900 Fermo, Italy; giovanni.cangelosi@virgilio.it

**Keywords:** hematopoietic cell transplantation (HCT), malnutrition, nutritional status, Malnutrition Universal Screening Tool (MUST), Global Leadership Initiative on Malnutrition (GLIM)

## Abstract

Background/Objectives: Hematopoietic cell transplantation (HCT) is a curative treatment for various hematological diseases but can lead to complications which increase malnutrition risk, particularly in allogeneic transplantation patients. This study evaluates the nutritional status evolution of patients undergoing HCT during hospitalization and follow-up. Methods: This retrospective observational study included 365 patients, divided into two groups: 134 underwent allogeneic HCT, while 231 underwent autologous transplantation or CAR-T therapy. Nutritional status was evaluated using Body Mass Index (BMI), Malnutrition Universal Screening Tool (MUST), and Global Leadership Initiative on Malnutrition (GLIM) criteria at four-time points: hospital admission, discharge, two-week follow-up, and one-month follow-up. Non-relapse-related complications were assessed based on hospital readmissions and reports during follow-up visits. Results: Patients experienced significant nutritional deterioration, with decreases in Body Mass Index (BMI) (*p* < 0.001) and increases in Malnutrition Universal Screening Tool (MUST) (*p* < 0.001) and Global Leadership Initiative on Malnutrition (GLIM) scores (*p* < 0.001), particularly among allogeneic transplant recipients (*p* = 0.025). Severe malnutrition or high malnutrition risk at discharge correlated with increased hospital readmissions during the follow-up (*p* = 0.024). Conclusions: The observed decline in nutritional status and its associated complications highlight the necessity of multidisciplinary interventions, such as nutritional prehabilitation programs and nutritional support protocols, to enhance clinical outcomes and reduce complications in HCT patients.

## Simple Summary

Hematopoietic cell transplantation (HCT) is a treatment for severe hematological disorders that can be performed as autologous, allogeneic, or chimeric antigen receptor T cells (CAR-T) therapy. While effective, these treatments often lead to nutritional challenges due to chemotherapy, immunosuppression, and complications like graft-versus-host disease. Malnutrition is linked to poorer recovery and increased hospital readmissions. This study evaluates the nutritional status of patients undergoing transplantation, aiming to identify those at risk of complications and understand how malnutrition develops over time. By using comprehensive screening tools, this research highlights the importance of nutritional support before and after transplantation. The findings suggest that early interventions could improve patient outcomes, reduce complications, and provide valuable insights for clinical guidelines. This research emphasizes the role of nutrition in recovery and offers a foundation for multidisciplinary approaches to patient care.

## 1. Introduction

Hematopoietic cell transplantation (HCT) is a potentially curative treatment for hematological malignancies such as leukemia, lymphoma, and myeloma [1]. Depending on the clinical indications, patients may undergo autologous transplantation, allogeneic transplantation [2], or chimeric antigen receptor T cells (CAR-T) therapy [3]. Each modality presents unique characteristics.

In autologous HCT, the patient’s hematopoietic cells (HCs) are mobilized, collected through leukapheresis, and cryopreserved until reinfusion after a conditioning regimen. This approach minimizes the immunological complications, but the pancytopenic phase and immunosuppression pose risks such as infections and organ toxicities [4,5].

Allogeneic HCT involves the infusion of HCs from an external donor, selected based on HLA compatibility [6]. While the graft-versus-tumor effect enhances the therapeutic efficacy [7], it also increases the risk of graft-versus-host disease (GVHD), a significant complication characterized by immune-mediated damage to the gastrointestinal tract, liver, and skin [8].

CAR-T therapy entails engineering the patient’s T cells to express a chimeric antigen receptor (CAR), enabling the targeted elimination of tumor cells [3,9]. While highly effective, this therapy is associated with unique toxicities, including cytokine release syndrome and neurotoxicity, which require careful management [10].

Across all HCT modalities, nutritional challenges are significant and can negatively affect patient outcomes [11]. High-dose chemotherapy and radiation used during conditioning regimens for autologous and allogenic transplantation frequently result in mucositis, nausea, vomiting, and decreased nutrient intake, exacerbating the risk of malnutrition [12]. In the case of allogeneic HTC, the risk of malnutrition is exacerbated by a longer aplastic period compared to other modalities and by the potential development of graft-versus-host disease (GVHD). Acute GVHD affects epithelial tissues, the liver, and the gastrointestinal tract, leading to profuse diarrhea and protein losses through the mucosa, further worsening caloric depletion. Additionally, chronic complications such as chronic GVHD, often associated with opportunistic and respiratory infections, may contribute to prolonged nutritional impairment [13,14,15,16,17]. Patients undergoing CAR-T therapy also face nutritional challenges. The risk of cytokine release syndrome (CRS) and neurotoxicity can compromise nutritional status. Symptoms such as fever, hypotension, and hypoxia during CRS, along with cognitive disturbances or encephalopathy, may negatively affect food intake and the overall nutritional management of the patient [10]. These factors underline the importance of accurate nutritional assessment and monitoring throughout the HCT process [18,19].

Despite the availability of numerous tools for nutritional assessment, there is currently no unified international consensus on the optimal method for patients undergoing HCT [20,21]. Each assessment approach has inherent limitations, particularly with regard to diagnostic specificity and the predictive value of clinical outcomes [22]. Commonly used serum markers, such as albumin and prealbumin, although practical for routine screening, are significantly influenced by systemic inflammatory processes, which can lead to their misinterpretation as indicators of nutritional status, especially in critically ill patients or those undergoing HCT [8]. Furthermore, these markers do not provide a full picture of a patient’s nutritional health, as they fail to account for factors such as muscle mass or functional status, which are crucial in the assessment of nutritional risk. The criteria of the Global Leadership Initiative on Malnutrition (GLIM), when combined with established screening tools such as the Malnutrition Universal Screening Tool (MUST), provide a more robust and comprehensive diagnostic approach. By integrating clinical, anthropometric, and functional data, these combined tools offer a more accurate assessment [23]. However, even these methods have limitations, including the challenge of accurately measuring certain anthropometric variables in hospitalized or severely ill patients and the subjective nature of clinical judgments. These constraints highlight the ongoing need for more precise and universally accepted tools for assessing malnutrition, particularly in high-risk populations like those undergoing HCT. This multidimensional methodology could represent a significant advancement in standardizing nutritional assessments for this complex patient population, enhancing diagnostic accuracy and potentially optimizing therapeutic interventions [24].

### Study Objectives

The aim of this study is to evaluate the nutritional status evolution of patients undergoing HCT during hospitalization and follow-up, using tools such as BMI, MUST, and the GLIM criteria. By addressing the multifaceted nature of malnutrition in this context, this research aims to mitigate non-relapse-related complications and improve long-term clinical outcomes.

## 2. Materials and Methods

### 2.1. Ethical Considerations

The study was conducted following Good Clinical Practice guidelines, the current version of the Declaration of Helsinki, and national regulations. The relevant Independent Ethics Committee reviewed and approved the protocol, titled “Nut 24.01”, and its appendices.

### 2.2. Setting

This study was conducted at the Bone Marrow Transplant Unit of a tertiary hospital in Northern Italy, Italy.

### 2.3. Study Population

The sample initially considered included 378 patients, of whom 13 were excluded due to incomplete clinical documentation. Consequently, a total of 365 patients were included (approximately 120 per year), divided into two cohorts: 134 patients underwent allogeneic transplantation (approximately 40 per year), and 231 patients underwent autologous transplantation or CAR-T therapy (approximately 80 per year).

*Inclusion Criteria:* Patients who underwent HCT at IRCCS Humanitas Research Hospital between January 2020 and December 2022; patients aged 18 years or older.

*Exclusion Criteria:* Patients without nutritional status documentation; patients receiving enteral or parenteral nutrition at baseline (T0).

### 2.4. Study Design

This was a retrospective observational study. Data were extracted from primary sources, including electronic medical records, follow-up visits or calls, and emergency department access logs. Data were anonymized and coded, with each patient assigned a unique ID to correlate data across sources. Patients were stratified into two cohorts: one comprising those who underwent allogeneic transplantation, and the other including those who underwent autologous transplantation or CAR-T therapy. As reported in the literature, allogeneic transplant recipients are at higher risk of malnutrition. Additional analyses were conducted based on the type of transplantation. Nutritional status risk was assessed using the MUST [25], while nutritional status itself was evaluated using BMI values and malnutrition diagnoses based on the GLIM criteria [26]. These tools were retrospectively reconstructed using data from electronic medical records; patients with incomplete or missing data were excluded. Evaluations were performed at four time points: T0: upon hospital admission; T1: at hospital discharge; T2: two weeks’ post-discharge follow-up; T3: one-month post-discharge follow-up. In particular, for MUST scoring, the oncological condition was not considered an “active acute disease” at baseline (T0). At T1, acute disease status was limited to the presence of GVHD, as other complications were reported as resolved in discharge summaries. Complications arising during the study periods were monitored through daily vital sign recordings and reviews of medical and nursing records during hospitalization, as well as reports from readmissions, outpatient visits, or phone consultations during the follow-up. Complications were graded using the Common Terminology Criteria for Adverse Events [27] whenever applicable.

Potential confounding variables were also considered, specifically the presence of diabetes mellitus or dietary restrictions, including previous surgeries impacting nutrient absorption, food allergies, inflammatory bowel diseases, and celiac disease. Specifically, only these variables were assessed because the retrospective nature of the data did not allow for a complete assessment of other potential comorbidities, which could have introduced potential biases.

During the period under consideration, no specific nutritional treatment protocols were in place. Consequently, the nutritional treatment was managed by physicians based on the specific nutritional needs of the patients, taking into account their individual nutritional status and adapting the approach to each clinical case.

### 2.5. Statistical Analysis

Data were described as counts and percentages for categorical variables, or as mean and standard deviation for approximately Gaussian continuous variables. If data were not normally distributed, median values were also reported. Normality was assessed using the Shapiro–Wilk test. The prevalence of complications in each group was expressed as counts, percentages, and 95% confidence intervals. The associations with initial MUST and GLIM scores were analyzed using logistic regression. Positive associations were adjusted for potential confounders. Logistic regression analysis was also used to evaluate the association of non-relapse-related complications with MUST and GLIM scores. Independent variables with a *p*-value below 0.2 were included as confounders. The associations between MUST and GLIM scores and hospital readmissions were assessed using survival analysis, with the first readmission as the event of interest. Positive associations were adjusted for confounders. Additionally, the association of MUST and GLIM scores with the number of readmissions within one month of transplantation was evaluated. All analyses were performed separately for each stratification group using Stata version 18 or higher. The significance threshold was set at 0.05.

## 3. Results

### 3.1. Study Population Analysis

This study included adult patients who underwent HCT between January 2020 and December 2022 (Table 1). A total of 365 patients of both sexes were included, with a predominance of males (59.2%) and a mean age of 54 years (54.3 ± 13.4 years). The underlying diseases leading to transplantation were grouped into five main categories: leukemia (17.5%), Hodgkin lymphoma (11%), non-Hodgkin lymphoma (41.4%), myeloma (27.1%), and other (3%). The “other” category included conditions that did not fit the aforementioned groups. The mean hospital stay was 24 days, with a longer duration observed for patients undergoing allogeneic transplantation (31 days, CI = 16 to 125; *p* < 0.001). BMI values were reconstructed for all time points analyzed. At admission, the mean BMI of the sample population was 25 kg/m^2^ (25.3 ± 4.5 kg/m^2^).

### 3.2. Observation of the Nutritional Status Evolution

**Figure 1:**
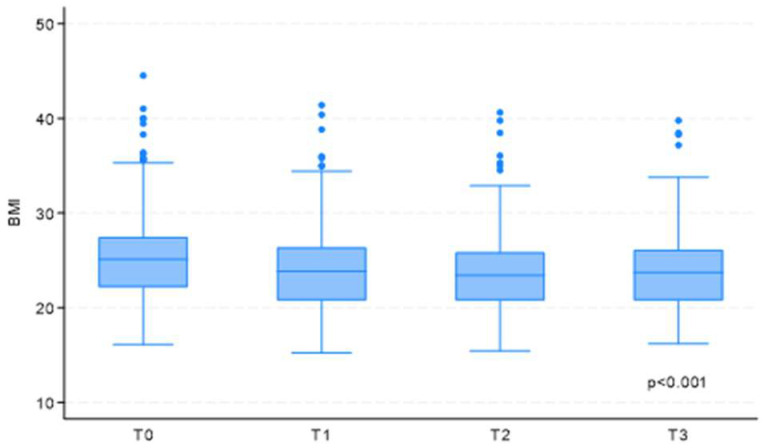
BMI variation over time. Legend: *p*: *p*-value; BMI: Body Mass Index; T0: upon hospital admission; T1: at hospital discharge; T2: two weeks post-discharge follow-up; T3: one-month post-discharge follow-up.

The analysis revealed a statistically significant decrease in the Body Mass Index (BMI) of the sample population across the four time points analyzed (coefficient −0.48; 95% CI = −0.53 to −0.42; *p* < 0.001), with the most pronounced difference observed between T0 and T1 (coefficient −1.30; 95% CI = −1.44 to −1.16; *p* < 0.001) (Figure 1).

Patients were divided into two cohorts: the first included those who underwent autologous transplantation or CAR-T therapy, while the second comprised patients who underwent allogeneic transplantation. In both groups, BMI values continued to vary over time (coefficient −0.48; 95% CI = −0.53 to −0.42; *p* < 0.001), with a more pronounced decrease observed among patients who underwent allogeneic transplantation (coefficient −0.57; 95% CI = −1.02 to −0.16; *p* = 0.012).

**Figure 2:**
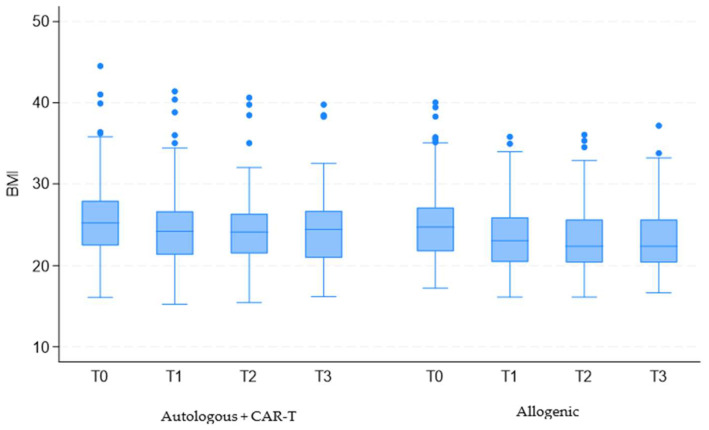
Evolution of BMI over time for patients grouped into two cohorts. Legend: CAR-T: chimeric antigen receptor T cells; BMI: Body Mass Index; T0: upon hospital admission; T1: at hospital discharge; T2: two weeks post-discharge follow-up; T3: one-month post-discharge follow-up.

The period showing the most significant reduction in BMI was between hospital admission (T0) and discharge (T1) (coefficient −1.30; 95% CI = −1.44 to −1.17; *p* < 0.001). This analysis also highlighted a more substantial difference among patients who underwent allogeneic transplantation (coefficient −0.62; 95% CI = −1.16 to −0.08; *p* = 0.025) (Figure 2).

BMI values were analyzed and adjusted for potential confounding variables such as a diagnosis of diabetes mellitus or the presence of dietary restrictions. BMI values continued to decrease over time (coefficient −0.48; 95% CI = −0.53 to −0.42; *p* < 0.001), with a more pronounced reduction in patients undergoing allogeneic transplantation (coefficient −0.52; 95% CI = −0.97 to −0.07; *p* = 0.025). Patients with diabetes mellitus showed higher BMI values (coefficient 2.68; 95% CI = 0.77 to 4.58; *p* = 0.006), while the presence of dietary restrictions was not significant (coefficient −0.59; 95% CI = −1.72 to 0.54; *p* = 0.302).

Focusing on the period between T0 and T1, the BMI reduction remained statistically significant (coefficient −1.30; 95% CI = −1.44 to −1.17; *p* < 0.001), with a greater decrease observed among patients undergoing allogeneic transplantation (coefficient −0.55; 95% CI = −1.10 to −0.07; *p* = 0.047). Patients with diabetes mellitus maintained higher BMI values (coefficient 2.71; 95% CI = 0.76 to 4.66; *p* = 0.006), while dietary restrictions did not show significant variations (coefficient −0.52; 95% CI = −1.71 to 0.68; *p* = 0.394).

Among patients with diabetes mellitus, the BMI decrease remained statistically significant (coefficient −0.47; 95% CI = −0.68 to −0.26; *p* < 0.001), but the effect of cohort stratification disappeared (coefficient 2.34; 95% CI = −0.94 to 5.63; *p* = 0.162), as the number of diabetic patients undergoing allogeneic transplantation was reduced to only six individuals.

In patients without a diagnosis of diabetes mellitus, a significant decrease in BMI values was still observed (coefficient −0.48; 95% CI = −0.53 to −0.42; *p* < 0.001), with a more marked loss in allogeneic transplantation patients (coefficient −0.59; 95% CI = −1.05 to −0.14; *p* = 0.010).

**Figure 3:**
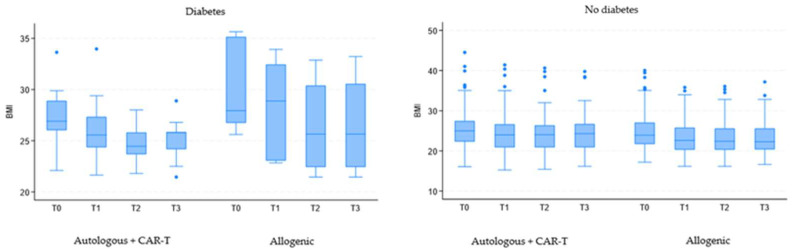
Evolution of BMI over time for patients grouped by diabetes mellitus diagnosis. Legend: CAR-T: chimeric antigen receptor T cells; BMI: Body Mass Index; T0: upon hospital admission; T1: at hospital discharge; T2: two weeks post-discharge follow-up; T3: one-month post-discharge follow-up.

The same pattern was maintained when limiting the analysis to the T0 to T1 interval. In diabetic patients, the BMI variation over time remained relevant (coefficient −1.18; 95% CI = −1.68 to −0.68; *p* < 0.001), but the group stratification lost significance (coefficient 2.71; 95% CI = 0.55 to 5.98; *p* = 0.104). Non−diabetic patients retained significant BMI reductions over time (coefficient −1.31; 95% CI = −1.45 to −1.17; *p* < 0.001) and in cohort stratification (coefficient −0.65; 95% CI = −1.20 to −0.10; *p* = 0.020) (Figure 3).

Finally, regarding the evaluation of BMI, the progression over time was analyzed by dividing patients based on the type of transplantation. A general progressive reduction was observed (coefficient −0.47; 95% CI = −0.53 to −0.42; *p* < 0.001). Comparing individuals who underwent autologous transplantation with those who underwent allogeneic transplantation revealed a significant difference (coefficient 1.11; 95% CI = 0.51 to 1.71; *p* < 0.001). However, no significant difference was found when comparing CAR-T therapy patients with those treated via allogeneic transplantation (coefficient 0.23; 95% CI = −0.29 to 0.75; *p* = 0.388).

**Figure 4:**
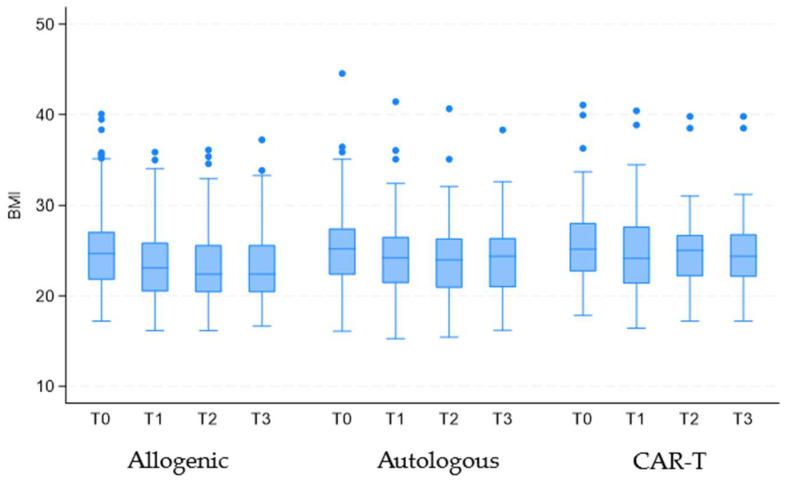
Evolution of BMI over time for patients grouped by transplantation type. Legend: CAR-T: chimeric antigen receptor T cells; BMI: Body Mass Index; T0: upon hospital admission; T1: at hospital discharge; T2: two weeks post-discharge follow-up; T3: one-month post-discharge follow-up.

A similar trend was observed when focusing the analysis on the T0 to T1 interval, which showed the most significant BMI reduction (coefficient −1.30; 95% CI = −1.43 to −1.17; *p* < 0.001). The difference remained significant when comparing autologous and allogeneic transplantation (coefficient 1.17; 95% CI = 0.51 to 1.83; *p* < 0.001) but not between CAR-T therapy and allogeneic transplantation (coefficient 0.11; 95% CI = −0.53 to 0.75; *p* = 0.737). These differences are likely due to the distinct clinical challenges faced by patients undergoing autologous versus allogeneic transplantation. Allogeneic transplantation is associated with a stronger immune response and a higher incidence of complications such as GVHD, which can lead to more pronounced weight loss and nutritional decline compared to autologous transplantation, where the immune response is less intense (Figure 4).

Considering the reconstructed scales, the evolution of MUST scores was analyzed, revealing a general progressive increase over time (coefficient 0.32; 95% CI = 0.28 to 0.35; *p* < 0.001), with the most pronounced increase occurring between patient admission and discharge (coefficient 0.73; 95% CI = 0.65 to 0.81; *p* < 0.001).

When dividing patients into groups based on transplantation type, comparing those who underwent allogeneic transplantation with individuals treated with the other two types, the progressive increase was maintained (coefficient 0.32; 95% CI = 0.28 to 0.35; *p* < 0.001), with a greater increase observed in the allogeneic group (coefficient 0.24; 95% CI = 0.12 to 0.36; *p* < 0.001). The most significant increase also occurred between T0 and T1 (coefficient 0.73; 95% CI = 0.65 to 0.81; *p* < 0.001), with a higher degree of scale increase in allogeneic transplantation patients (coefficient 0.12; 95% CI = −0.01 to 0.24; *p* = 0.063).

Adjusting for confounding variables revealed no significant changes related to the presence of diabetes mellitus (coefficient −0.19; 95% CI = −0.45 to 0.06; *p* = 0.137) or dietary restrictions (coefficient −0.01; 95% CI = −0.21 to 0.19; *p* = 0.914).

When patients were grouped by transplantation type, MUST scores continued to increase over time (coefficient 0.32; 95% CI = 0.28 to 0.35; *p* < 0.001). Specifically, a significant difference was observed both when comparing autologous transplantation patients with those undergoing allogeneic transplantation (coefficient −0.21; 95% CI = −0.34 to −0.09; *p* = 0.001) and when comparing CAR−T therapy patients with allogeneic transplantation patients (coefficient −0.30; 95% CI = −0.46 to −0.13; *p* < 0.001).

The interval showing the most significant increase remained between patient admission and discharge (coefficient 0.73; 95% CI = 0.65 to 0.81; *p* < 0.001), with differences persisting between patients undergoing allogeneic transplantation and those treated with the other two types of transplantation (coefficient autologous vs. allogeneic −0.12; 95% CI = −0.25 to 0.01; *p* = 0.075; coefficient CAR−T vs. allogeneic −0.10; 95% CI = −0.28 to 0.07; *p* = 0.226) (Table 2).

For the diagnosis of malnutrition, the GLIM criteria were analyzed. To quantify the collected data, a score of 0 was assigned for the absence of malnutrition, 1 point for moderate malnutrition, and 2 points for severe malnutrition. The analysis revealed a progressive increase in scores over time (coefficient 0.14; 95% CI = 0.11 to 0.16; *p* < 0.001). For the GLIM criteria, the interval between T0 and T1 showed an increase in values, albeit with a smaller variation compared to previous analyses using BMI and the MUST scale (coefficient 0.03; 95% CI = 0.01 to 0.05; *p* = 0.009).

When dividing patients into two cohorts, one including those who underwent allogeneic transplantation and the other comprising the individuals who received the other two transplantation types, a progressive increase in the degree of malnutrition was observed (coefficient 0.13; 95% CI = 0.11 to 0.16; *p* < 0.001), with a greater increase among patients in the first group (coefficient 0.20; 95% CI = 0.14 to 0.26; *p* < 0.001). This trend persisted, with numerically smaller but statistically significant results, during the interval between hospital admission and discharge (time coefficient 0.03; 95% CI = 0.01 to 0.06; *p* = 0.008; allogeneic vs. non-allogeneic coefficient 0.04; 95% CI = 0.02 to 0.07; *p* = 0.001).

The values were adjusted for the presence of diabetes mellitus (diabetes coefficient −0.03; 95% CI = −0.14 to 0.09; *p* = 0.669) and dietary restrictions (dietary restrictions coefficient −0.03; 95% CI = −0.12 to 0.06; *p* = 0.499), neither of which were statistically significant.

Further analyses were conducted by stratifying patients by transplantation type. This also revealed an increase in malnutrition status over time (coefficient 0.13; 95% CI = 0.11 to 0.16; *p* < 0.001). Patients undergoing autologous transplantation showed a greater increase in GLIM scores compared to the other two types (autologous vs. allogeneic coefficient −0.19; 95% CI = −0.25 to −0.13; *p* < 0.001; CAR-T vs. allogeneic coefficient −0.22; 95% CI = −0.30 to −0.14; *p* < 0.001). Even with this stratification, the difference between T0 and T1 remained small (coefficient 0.03; 95% CI = 0.01 to 0.06; *p* = 0.008), and the difference between transplantation types during the analyzed interval also showed a reduced delta value (autologous vs. allogeneic coefficient −0.04; 95% CI = −0.07 to −0.02; *p* = 0.001; CAR-T vs. allogeneic coefficient −0.04; 95% CI = −0.08 to −0.01; *p* = 0.018) (Table 3).

### 3.3. Evaluation of the Complication Onset

This study analyzed the onset of complications not related to the relapse of onco-hematological diseases during the time intervals considered, specifically between hospital admission and discharge. Where possible, these complications were assessed using the Common Terminology Criteria for Adverse Events [25].

During the period between T0 and T1, complications that could potentially reduce caloric intake or cause malabsorption in patients were evaluated. The analysis revealed a lower incidence of mucositis in patients undergoing CAR-T therapy (*p* = 0.005) compared to the other transplantation types, while patients who underwent autologous transplantation were more frequently affected by this complication.

Similarly, patients receiving CAR-T therapy demonstrated a lower incidence of diarrhea, whereas no significant differences were observed between the other two transplantation types (*p* = 0.346). Regarding impaired liver function tests, allogeneic transplantation patients exhibited this symptom more frequently, while no relevant differences were found between patients undergoing autologous transplantation or CAR-T therapy (*p* = 0.658) (Table 4).

Among patients undergoing allogeneic transplantation, the prevalence of GVHD was evaluated at different time points post-procedure. At T1, 9 out of 134 patients (6.72%) were diagnosed with GVHD. This prevalence increased notably at T2, with 28 out of 124 patients (22.58%) affected. By T3, the prevalence declined slightly to 22 out of 124 patients (17.74%).

The potential association between GVHD and a greater reduction in BMI values during the periods following its onset was analyzed. However, no statistically significant differences were observed. At T1, the change in BMI from T2 to T1 was −0.11 ± 0.69 for patients with GVHD (n = 6) compared to 0.00 ± 0.86 for those without GVHD (n = 99, *p* = 0.743). At T2, the BMI change from T3 to T2 was −0.09 ± 0.80 for patients with GVHD (n = 25) versus −0.09 ± 0.88 for those without GVHD (n = 79, *p* = 0.963).

To evaluate the association between Body Mass Index (BMI) at discharge (T1) and the development of complications, the population was stratified into four categories: underweight patients (BMI < 18.5 kg/m^2^), normal-weight patients (BMI 18.5–25 kg/m^2^), overweight patients (BMI 25–30 kg/m^2^), and obese patients (BMI > 30 kg/m^2^). The analysis indicated a potential predisposition for underweight patients to develop complications at T3 (*p* = 0.030) but not at T2 (*p* = 0.296).

Although a higher number of hospital readmissions was observed among obese individuals during the first follow-up (T2) (*p* = 0.042), this difference was no longer statistically significant at T3 (*p* = 0.712). Considering hospital readmissions throughout the follow-up period (at least one readmission at T2 or T3), none of the groups showed data significantly differing from the others (*p* = 0.356) (Table 5).

Analyzing the association between a higher MUST score and the development of complications during the follow-up period did not reveal significant results. However, an increased incidence of hospital readmissions (*p* = 0.044) was observed at least once during the two follow-up time points (T2 and T3) among patients with a higher risk of malnutrition (MUST ≥ 2) at discharge (T1) (Table 6).

Finally, the correlation between the diagnosis of malnutrition in patients, assessed using the GLIM criteria at discharge (T1), and the development of complications during subsequent periods was investigated. The comparison did not reveal significant differences regarding the complications developed. However, an increase in hospital readmissions at least once during the two follow-up periods (T2 and T3) was observed among the patients classified as having “severe” malnutrition (*p* = 0.024), although the sample size was small. Most patients assessed using the scale at discharge (T1) were classified as “not malnourished” (Table 7).

## 4. Discussion

In this study, the primary objective was to evaluate the nutritional status of an adult population of patients undergoing HCT. Multiple parameters were used for this purpose, as no unanimous international consensus exists regarding the most suitable methods [23]. The MUST scale was reconstructed using clinical chart data to assess malnutrition risk in association with the GLIM criteria for diagnosing malnutrition, a combination that has shown good outcomes in recent studies [24]. Additionally, BMI was considered, as in previous studies with comparable investigations [8].

The investigation demonstrated a progressive deterioration in the nutritional status of all patients over time, as evidenced by a decrease in mean BMI, an increase in MUST scores, and a higher number of patients classified as malnourished according to the GLIM criteria. When dividing the population into two cohorts, the first comprising patients undergoing allogeneic transplantation and the second including those who underwent autologous transplantation or CAR-T therapy, this deterioration was more significant among the first group, consistent with findings in the literature focusing on allogeneic transplantation patients [12].

A closer look at the analyses revealed that the most significant differences occurred between T0 and T1 for all parameters evaluated. Further stratification by transplantation type showed that in terms of the GLIM and MUST criteria, patients undergoing allogeneic transplantation demonstrated worse outcomes than those receiving autologous transplantation or CAR-T therapy. However, when considering BMI, the difference between allogeneic and autologous transplantations persisted, but the difference between allogeneic and CAR-T therapy did not. These results align with the international literature, which has shown a frequent predisposition of allogeneic transplantation patients to malnutrition episodes, likely due to the increased risk of developing infections [13]. The same article also attributed the worsening nutritional status in this population to the potential development of GVHD. In this study sample, however, no significant BMI reductions were observed in the time intervals following the onset of the disease, likely due to the small number of patients who developed the condition within the analyzed periods.

In this study, dietary restrictions in some patients proved to be an irrelevant factor concerning nutritional status. However, when adjusting for a prior diagnosis of diabetes mellitus, a milder reduction in BMI values was observed, while the differences remained consistent across all stratifications.

The literature [28] demonstrates an association between malnutrition and worse clinical outcomes. Similarly, to other studies, this research also aimed to evaluate the onset of complications unrelated to hematological disease relapse as a secondary objective. The data showed an association between severe malnutrition at discharge (T1), as assessed by the GLIM criteria, or a high risk of malnutrition according to the MUST scale, and an increase in hospital readmissions during at least one of the two follow-up periods analyzed (T2–T3).

These results, as confirmed by the literature [13], indicate a clinical deterioration in patients that, consistent with the findings of Cioce et al. [11], negatively impacts their quality of life. Similarly, as reported in a previous study [29], obese patients (BMI ≥ 30 kg/m^2^) were more likely to experience hospital readmissions during T2 compared to their normal-weight counterparts. These findings are consistent with the existing literature, which highlights the significant impact of nutritional status on post-transplant outcomes. Underweight patients face an elevated risk of infections, delayed recovery, and increased morbidity, while obesity has been associated with prolonged hospital stays and higher readmission rates [29,30,31]. In our analysis, underweight patients showed a predisposition to complications at T3, whereas obese individuals experienced higher readmission rates during the early follow-up period, consistent with these trends. Moreover, the role of malnutrition, assessed using the MUST and GLIM criteria, is well-documented in the literature as a significant predictor of adverse outcomes in hematological patients undergoing intensive treatments [32,33]. Our results similarly highlighted an association between severe malnutrition and increased hospital readmissions, reinforcing the importance of early nutritional assessment and intervention to improve post-transplant outcomes.

This study emphasizes the importance of a multidisciplinary approach in managing hematological patients with nutritional status alterations. A team comprising various healthcare professionals allows for comprehensive patient care, addressing the multiple factors influencing their health. Integrating diverse expertise is crucial for implementing early protocols of nutritional support, as is already done for other neoplastic diseases [34,35], to prevent malnutrition and improve clinical outcomes. In this context, specialized healthcare professionals in nutritional care would play a key role in supporting both physicians and nutritionists. Thanks to their ability to perform multidimensional patient assessments [36], these professionals are particularly suited to monitoring and supporting patients throughout their nutritional journey, ensuring continuous and personalized care while enhancing the collaborative efforts of the multidisciplinary team. Their presence not only enables the early identification of malnutrition signs and symptoms but also facilitates patient and family education on appropriate dietary practices. The introduction of specialized nurses, supported by the literature [37], represents an effective strategy for preventing malnutrition episodes, reducing hospital readmissions, and improving patients’ quality of life. This collaboration among various professionals not only optimizes clinical management but also contributes to a more efficient allocation of healthcare resources.

### Strengths and Limitations of the Study

This research presents several strengths. First, it is one of the first studies to investigate the evolution of nutritional status in patients undergoing HCT, recruiting a large sample size and stratifying the research by transplantation type over a three-year period. The analyses covered an extended time frame for each patient, not limited to the hospitalization period but including two follow-up evaluations at two weeks and one-month post-discharge. Additionally, conducting this study in a single center provided a consistent approach to patient care and data collection, reducing variability introduced by differences in institutional protocols. This monocentric design ensured uniformity in the follow-up procedures and documentation practices, thereby strengthening the reliability and internal validity of the findings. Moreover, the analytical methods employed in this study align with the literature, yielding clear and comparable results.

This study has several limitations that should be considered when interpreting its findings. First, the retrospective design of the study restricted the ability to include all desired parameters, especially during the follow-up period. Not all patients completed the full treatment pathway due to various disease-related or logistical factors, leading to incomplete data for some time points. Second, the research was conducted at a single hospital, which limits the geographical diversity and generalizability of the results. Furthermore, this study did not account for the different hematological conditions that led to various HCTs, nor did it consider the specific therapeutic regimens used. These factors may have influenced the outcomes, and future studies should aim to include a broader range of conditions and treatments for more comprehensive results. Another limitation is the lack of a standardized nutritional treatment protocol during the study period, which could have led to variability in the nutritional interventions provided to participants. Furthermore, the time points selected for nutritional status assessment, although chosen with considerable effort, were too close to therapeutic procedures; therefore, a longer follow-up would have been beneficial, but the retrospective nature of the data made it difficult to obtain this without losing substantial amounts of data over time. Finally, while non-recurrence-related complications were documented, their classification was limited by the information available in the patients’ medical records. In particular, infections, a significant non-recurrence-related complication, were not analyzed in detail due to insufficient data, but their inclusion in future prospective studies could provide valuable information.

## 5. Conclusions

This study examined the nutritional status of an adult population of patients undergoing HCT, highlighting a progressive deterioration in nutritional indicators. The worsening of nutritional status was particularly pronounced during the hospitalization phase, specifically between patient admission before the procedure and discharge. Notably, patients undergoing allogeneic transplantation showed a greater predisposition to malnutrition compared to those undergoing autologous transplantation or CAR-T therapy.

Furthermore, a correlation was observed between severe malnutrition or a high risk of malnutrition at discharge and an increase in hospital readmissions during at least one of the two follow-up periods considered. To improve outcomes for these patients, future studies could explore the effectiveness of implementing a multidisciplinary nutritional prehabilitation program. Such a program should ensure early intervention by a dedicated nutritional team and establish a continuum of care from the initial assessment through to follow-up. This approach could integrate standardized nutritional protocols to prevent malnutrition and its associated complications effectively.

## Figures and Tables

**Table 1:** Study population.

Variable	All	Allogenic		Autologous		CAR-T		*p*
		n		n		n		
Age (years)	54.3 ± 13.4	134	53.5 ± 14.2	173	54.8 ± 12.9	58	55.0 ± 12.6	0.837
Sex (male)	216 (59.2%)	134	80 (59.7%)	173	102 (59.0%)	58	34 (58.2%)	0.987
Disease		134		173		58		
Leukemia	64 (17.5%)		63 (47.0%)		0		1 (1.7%)	
Hodgkin Lymphoma	40 (11.0%)		15 (11.2%)		24 (13.9%)		1 (1.7%)	
Non-Hodgkin Lymphoma	151 (41.4%)		30 (22.4%)		65 (37.6%)		56 (96.6%)	
Myeloma	99 (27.1%)		24 (17.9%)		75 (43.4%)		0	
Other	11 (3.0%)		2 (1.5%)		9 (5.2%)		0	
Hospital Stay (days)	24 (7–125)	134	31 (16–125)	173	22 (7–61)	58	18.5 (15–65)	<0.001
Diabetes	24 (6.58%)	134	6 (4.48%)	173	14 (8.09%)	58	4 (6.90%)	0.467
BMI (kg/m^2^)								
T0	25.3 ± 4.5	134	25.2 ± 4.7	173	25.2 ± 4.3	58	25.8 ± 5.0	
T1	24.0 ± 4.3	132	23.7 ± 4.2	173	24.0 ± 4.2	58	24.8 ± 5.0	
T2	23.8 ± 4.2	106	23.2 ± 4.1	114	24.0 ± 4.0	36	25.0 ± 4.9	
T3	23.8 ± 4.2	104	23.2 ± 4.1	108	24.0 ± 4.0	35	25.0 ± 5.1	
MUST Score								
T0	0.30 ± 0.73	134	0.24 ± 0.60	173	0.34 ± 0.82	58	0.36 ± 0.72	
T1	1.16 ± 1.07	125	1.44 ± 1.15	171	1.02 ± 0.96	56	0.95 ± 1.10	
T2	1.89 ± 1.48	106	2.40 ± 1.43	114	1.68 ± 1.42	36	1.06 ± 1.26	
T3	1.59 ± 1.46	101	2.14 ± 1.50	108	1.30 ± 1.33	34	0.91 ± 1.14	

Legend: CAR-T: chimeric antigen receptor T cells; *p*: *p*-value; BMI: Body Mass Index; MUST: Malnutrition Universal Screening Tool; T0: upon hospital admission; T1: at hospital discharge; T2: two weeks post-discharge follow-up; T3: one-month post-discharge follow-up.

**Table 2:** Evolution of malnutrition risk over time.

MUST	n	Allogenic	n	Autologous	n	CAR-T	*p*
T0	134		173		58		0.697
0		113 (84.33%)		140 (80.92%)		44 (75.86%)	
1		11 (8.21%)		18 (10.40%)		8 (13.79%)	
>2		10 (7.46%)		15 (8.67%)		6 (10.34%)	
T1	125		171		56		0.004
0		28 (22.40%)		55 (32.16%)		24 (42.86%)	
1		44 (35.20%)		74 (43.27%)		19 (33.93%)	
>2		53 (42.40%)		42 (24.56%)		13 (23.21%)	
T2	106		114		36		<0.001
0		11 (10.38%)		26 (22.81%)		16 (44.44%)	
1		19 (17.92%)		31 (27.19%)		9 (25.00%)	
>2		76 (71.70%)		57 (50.00%)		11 (30.56%)	
T3	101		108		34		<0.001
0		15 (14.85%)		38 (35.19%)		17 (50.00%)	
1		22 (21.78%)		31 (28.70%)		8 (23.53%)	
>2		64 (63.37%)		39 (36.11%)		9 (26.47%)	

Legend: CAR-T: chimeric antigen receptor T cells; *p*: *p*-value; MUST: Malnutrition Universal Screening Tool; T0: upon hospital admission; T1: at hospital discharge; T2: two weeks post-discharge follow-up; T3: one-month post-discharge follow-up.

**Table 3:** Evolution of malnutrition diagnosis over time.

GLIM Criteria	n	Allogenic	n	Autologous	n	CAR-T	*p*
T0	134		173		58		
No		134 (100%)		173 (100%)		58 (100%)	
Moderate		0		0		0	
Severe		0		0		0	
T1	125		171		56		0.008
No		118 (94.40%)		171 (100%)		56 (100%)	
Moderate		3 (2.40%)		0		0	
Severe		4 (3.20%)		0		0	
T2	106		112		36		<0.001
No		59 (55.66%)		95 (84.82%)		32 (88.89%)	
Moderate		23 (21.70%)		6 (5.36%)		2 (5.56%)	
Severe		24 (22.64%)		11 (9.82%)		2 (2.56%)	
T3	101		108		34		0.001
No		68 (67.33%)		96 (88.89%)		31 (91.18%)	
Moderate		11 (10.89%)		4 (3.70%)		2 (5.88%)	
Severe		22 (21.78%)		8 (7.41%)		1 (2.94%)	

Legend: CAR-T: chimeric antigen receptor T cells; *p*: *p*-value; GLIM: Global Leadership Initiative on Malnutrition; T0: upon hospital admission; T1: at hospital discharge; T2: two weeks post-discharge follow-up; T3: one-month post-discharge follow-up.

**Table 4:** Onset of complications.

Variabile	All	Allogenic	Autologous	CAR-T	*p*
n	365	134	173	58	
Mucositis					<0.001
G0	95 (26.03%)	29 (21.64%)	14 (8.09%)	52 (89.66%)	
G1	43 (11.78%)	12 (8.96%)	25 (14.45%)	6 (10.34%)	
G2	82 (22.47%)	37 (27.61%)	45 (26.01%)	0	
G3	130 (35.62%)	48 (35.82%)	82 (47.40%)	0	
G4	15 (4.11%)	8 (5.97%)	7 (4.05%)	0	
Diarrhea					<0.001
G0	157 (43.01%)	41 (30.60%)	65 (37.57%)	51 (87.93%)	
G1	108 (29.59%)	43 (32.09%)	59 (34.10%)	6 (10.34%)	
G2	72 (19.73%)	37 (27.61%)	34 (19.65%)	1 (1.72%)	
G3	28 (7.67%)	13 (9.70%)	15 (8.67%)	0 (0.00%)	
G4	0	0	0	0	
Impaired liver function tests					<0.001
G0	319 (87.40%)	102 (76.12%)	162 (93.64%)	55 (94.83%)	
G1	21 (5.75%)	13 (9.70%)	7 (4.05%)	1 (1.72%)	
G2	10 (2.74%)	9 (6.72%)	1 (0.58%)	0 (0.00%)	
G3	13 (3.56%)	8 (5.97%)	3 (1.73%)	2 (3.45%)	
G4	2 (3.56%)	2 (1.49%)	0 (0.00%)	0 (0.00%)	

Legend: CAR-T: chimeric antigen receptor T cells; *p*: *p*-value; G: graded using the Common Terminology Criteria for Adverse Events [25].

**Table 5:** Correlation between Body Mass Index and complications.

	BMI (T1)				*p*
	<18.5	18.5–25	25–30	>30	
T2 (n)	29	187	101	27	
Complications T2	13 (44.8%)	84 (44.9%)	43 (42.6%)	17 (63.0%)	0.296
Readmissions T2	2 (6.9%)	12 (6.4%)	1 (0.99%)	3 (11.1%)	0.042
Readmissions T2 or T3	3 (10.3%)	21 (11.2%)	6 (59%)	4 (14.8%)	0.356
T3 (n)	26	187	99	27	
Complications T3	12 (46.2%)	62 (33.2%)	20 (20.2%)	9 (33.3%)	0.030
Readmissions T3	1 (3.9%)	13 (7.0%)	5 (5.1%)	3 (11.1%)	0.713

Legend: *p*: *p*-value; BMI: Body Mass Index; T1: at hospital discharge; T2: two weeks post-discharge follow-up; T3: one-month post-discharge follow-up.

**Table 6:** Correlation between malnutrition risk and complications.

	MUST (T1)			*p*
	0	1	≥2	
T2 (n)	104	135	105	
Complications T2	41 (39.4%)	64 (47.4%)	52 (49.5%)	0.297
Readmissions T2	2 (1.9%)	7 (5.2%)	9 (8.6%)	0.092
Readmissions T2 or T3	8 (7.7%)	9 (6.7%)	17 (16.2%)	0.044
T3 (n)	104	132	103	
Complications T3	29 (27.9%)	36 (27.3%)	38 (36.9%)	0.226
Readmissions T3	6 (5.8%)	6 (4.6%)	10 (9.7%)	0.287

Legend: *p*: *p*-value; MUST: Malnutrition Universal screening Tool; T1: at hospital discharge; T2: two weeks post-discharge follow-up; T3: one-month post-discharge follow-up.

**Table 7:** Correlation between malnutrition diagnosis and complications.

	GLIM (T1)			*p*
	n	Moderate	Severe	
T2 (n)	337	3	4	
Complications T2	154 (45.7%)	1 (33.3%)	2 (50.0%)	1.000
Readmissions T2	17 (5.0%)	1 (33.3%)	0	0.162
Readmissions T2 or T3	31 (9.2%)	1 (33.3%)	2 (50.0%)	0.024
T3 (n)	332	3	4	
Complications T3	98 (29.5%)	2 (66.7%)	3 (75.0%)	0.050
Readmissions T3	20 (6.0%)	0	2 (50.0%)	0.034

Legend: *p*: *p*-value; GLIM: Global Leadership Initiative on Malnutrition; T1: at hospital discharge; T2: two weeks post-discharge follow-up; T3: one-month post-discharge follow-up.

## Data Availability

The study data can be provided to the authors upon request.

## References

[1] Jessop H., Farge D., Saccardi R., Alexander T., Rovira M., Sharrack B., Greco R., Wulffraat N., Moore J., Kazmi M. (2019). General information for patients and carers considering haematopoietic stem cell transplantation (HSCT) for severe autoimmune diseases (ADs): A position statement from the EBMT Autoimmune Diseases Working Party (ADWP), the EBMT Nurses Group, the EBMT Patient, Family and Donor Committee and the Joint Accreditation Committee of ISCT and EBMT (JACIE). Bone Marrow Transplant..

[2] Ryan A.M., Prado C.M., Sullivan E.S., Power D.G., Daly L.E. (2019). Effects of weight loss and sarcopenia on response to chemotherapy, quality of life, and survival. Nutrition.

[3] Ahmad A. (2020). CAR-T Cell Therapy. Int. J. Mol. Sci..

[4] Ross L.A., Stropp L.M., Cohen J.A. (2024). Autologous Hematopoietic Stem Cell Transplantation to Treat Multiple Sclerosis. Neurol. Clin..

[5] Tomblyn M., Chiller T., Einsele H., Gress R., Sepkowitz K., Storek J., Wingard J.R., Young J.A., Boeckh M.J., Center for International Blood and Marrow Research (2009). Guidelines for preventing infectious complications among hematopoietic cell transplantation recipients: A global perspective. Biol. Blood Marrow Transplant..

[6] Kenyon M., Babic A. (2018). The European Blood and Marrow Transplantation Textbook for Nurses: Under the Auspices of EBMT.

[7] Bazinet A., Popradi G. (2019). A general practitioner’s guide to hematopoietic stem-cell transplantation. Curr. Oncol..

[8] El-Ghammaz A.M.S., Ben Matoug R., Elzimaity M., Mostafa N. (2017). Nutritional status of allogeneic hematopoietic stem cell transplantation recipients: Influencing risk factors and impact on survival. Support. Care Cancer.

[9] Sterner R.C., Sterner R.M. (2021). CAR-T cell therapy: Current limitations and potential strategies. Blood Cancer J..

[10] Brudno J.N., Kochenderfer J.N. (2019). Recent advances in CAR T-cell toxicity: Mechanisms, manifestations and management. Blood Rev..

[11] Cioce M., Botti S., Lohmeyer F.M., Galli E., Magini M., Giraldi A., Garau P., Celli D., Zega M., Sica S. (2022). Nutritional status and quality of life in adults undergoing allogeneic hematopoietic stem cell transplantation. Int. J. Hematol..

[12] Brotelle T., Lemal R., Cabrespine A., Combal C., Hermet E., Ravinet A., Bay J.O., Bouteloup C. (2018). Prevalence of malnutrition in adult patients previously treated with allogeneic hematopoietic stem-cell transplantation. Clin. Nutr..

[13] Price S., Kim Y. (2022). Body Composition Impacts Hematopoietic Stem Cell Transplant Outcomes in Both Autologous and Allogeneic Transplants: A Systematic Review. Nutr. Cancer.

[14] Naymagon S., Naymagon L., Wong S.Y., Ko H.M., Renteria A., Levine J., Colombel J.F., Ferrara J. (2017). Acute graft-versus-host disease of the gut: Considerations for the gastroenterologist. Nat. Rev. Gastroenterol. Hepatol..

[15] Zeiser R., Socié G., Blazar B.R. (2016). Pathogenesis of acute graft-versus-host disease: From intestinal microbiota alterations to donor T cell activation. Br. J. Haematol..

[16] Rezvani A.R., Storer B.E., Storb R.F., Mielcarek M., Maloney D.G., Sandmaier B.M., Martin P.J., McDonald G.B. (2011). Decreased serum albumin as a biomarker for severe acute graft-versus-host disease after reduced-intensity allogeneic hematopoietic cell transplantation. Biol. Blood Marrow Transplant..

[17] Ruutu T., Juvonen E., Remberger M., Remes K., Volin L., Mattsson J., Nihtinen A., Hägglund H., Ringdén O., Nordic Group for Blood and Marrow Transplantation (2014). Improved survival with ursodeoxycholic acid prophylaxis in allogeneic stem cell transplantation: Long-term follow-up of a randomized study. Biol. Blood Marrow Transplant..

[18] Lazarow H., Nicolo M., Compher C., Kucharczuk C.R., Stadtmauer E.A., Landsburg D.J. (2019). Nutrition-Related Outcomes for Autologous Stem Cell Transplantation Patients. Clin. Lymphoma Myeloma Leuk..

[19] Ren G., Zhang J., Li M., Yi S., Xie J., Zhang H., Wang J. (2017). Protein blend ingestion before allogeneic stem cell transplantation improves protein-energy malnutrition in patients with leukemia. Nutr. Res..

[20] Zhang Z., Wan Z., Zhu Y., Zhang L., Zhang L., Wan H. (2021). Prevalence of malnutrition comparing NRS2002, MUST, and PG-SGA with the GLIM criteria in adults with cancer: A multi-center study. Nutrition.

[21] Wang B., Yan X., Cai J., Wang Y., Liu P. (2013). Nutritional assessment with different tools in leukemia patients after hematopoietic stem cell transplantation. Chin. J. Cancer Res..

[22] Carreras E., Dufour C., Mohty M., Kröger N. (2019). The EBMT Handbook: Hematopoietic Stem Cell Transplantation and Cellular Therapies.

[23] Hu Y., Zhang C., Zou C., Yang H., Chen Y., Liang T. (2023). Anthropometric measures and physical examination could be used to assess phenotypic GLIM (Global leadership initiative on malnutrition) criteria in heart failure patients. Nutr. Metab. Cardiovasc. Dis..

[24] Lima J., Brizola Dias A.J., Burgel C.F., Bernardes S., Gonzalez M.C., Silva F.M. (2022). Complementarity of nutritional screening tools to GLIM criteria on malnutrition diagnosis in hospitalised patients: A secondary analysis of a longitudinal study. Clin. Nutr..

[25] Elia M. (2003). THE ‘MUST’ REPORT Nutritional Screening of Adults: A Multidisciplinary Responsibility Development and Use of the ‘Malnutrition Universal Screening Tool’ (‘MUST’) for Adults. BAPEN.

[26] Cederholm T., Jensen G.L., Correia M.I.T.D., Gonzalez M.C., Fukushima R., Higashiguchi T., Baptista G., Barazzoni R., Blaauw R., Coats A. (2019). GLIM criteria for the diagnosis of malnutrition—A consensus report from the global clinical nutrition community. Clin Nutr..

[27] (2017). Common Terminology Criteria for Adverse Events (CTCAE) Version 5.0. U.S. Department of Health and Human Services. https://ctep.cancer.gov/protocoldevelopment/electronic_applications/ctc.htm.

[28] Eglseer D., Bauer S., Huber-Kraßnitzer B., Greinix H. (2021). Malnutrition risk prior to hematopoietic stem cell transplantation predicts mortality in adults. Bone Marrow Transplant..

[29] Fuji S., Kim S.W., Yoshimura K., Akiyama H., Okamoto S., Sao H., Takita J., Kobayashi N., Mori S., Japan Marrow Donor Program (2009). Possible association between obesity and posttransplantation complications including infectious diseases and acute graft-versus-host disease. Biol. Blood Marrow Transplant..

[30] Shah P., Orens J.B. (2013). Impact of nutritional state on lung transplant outcomes: The weight of the evidence. J. Heart Lung Transplant..

[31] Szovati S., Morrison C.F., Couch S.C. (2023). Nutritional Status of Allogeneic Hematopoietic Stem Cell Transplant Recipients and Post-transplant Outcomes. Nutr Cancer..

[32] Zou Y., Xu H., Lyu Q., Weng M., Cui J., Shi H., Song C. (2023). Malnutrition diagnosed by GLIM criteria better predicts long-term outcomes for patients with non-Hodgkin’s lymphoma: A prospective multicenter cohort study. Hematol. Oncol..

[33] Yu X., Zhao J., Xu J., Li L., Dong J., Yuan W., Shi J., Kuang Z. (2024). Association of Dietary Profiles and Nutritional Status Among Patients with Hematologic Diseases: A Prospective Cohort Study. Blood.

[34] De Pasquale G., Mancin S., Matteucci S., Cattani D., Pastore M., Franzese C., Scorsetti M., Mazzoleni B. (2023). Nutritional prehabilitation in head and neck cancer: A systematic review of literature. Clin. Nutr. ESPEN.

[35] Pellegrinelli A., Mancin S., Brolese A., Marcucci S., Roat O., Morenghi E., Morales Palomares S., Cattani D., Lopane D., Dacomi A. (2024). Impact of Preoperative Malnutrition on Patients with Pancreatic Neoplasms Post-Duodenopancreatectomy: A Retrospective Cohort Study. Nutrients.

[36] Boeykens K., Van Hecke A. (2018). Advanced practice nursing: Nutrition Nurse Specialist role and function. Clin. Nutr. ESPEN.

[37] Mancin S., Pipitone V., Testori A., Ferrante S., Soekeland F., Sguanci M., Mazzoleni B. (2024). Clinical nurse specialists in nutrition: A systematic review of roles and clinical experiences. Int. Nurs. Rev..

